# Pulmonary Metastasectomy in Colorectal Cancer: updated analysis of 93 randomized patients – control survival is much better than previously assumed

**DOI:** 10.1111/codi.15113

**Published:** 2020-06-14

**Authors:** M. Milosevic, J. Edwards, D. Tsang, J. Dunning, M. Shackcloth, T. Batchelor, A. Coonar, J. Hasan, B. Davidson, A. Marchbank, S. Grumett, N.R. Williams, F. Macbeth, V. Farewell, T. Treasure

**Affiliations:** ^1^ Institute for Lung Diseases of Vojvodina Thoracic Surgery Clinic Sremska Kamenica Serbia; ^2^ Sheffield Teaching Hospitals NHS Foundation Trust Sheffield UK; ^3^ Basildon and Thurrock University Hospitals NHS Foundation Trust Basildon UK; ^4^ South Tees Hospitals NHS Foundation Trust The James Cook University Hospital Middlesbrough UK; ^5^ Liverpool Heart And Chest Hospital NHS Foundation Trust Liverpool UK; ^6^ Bristol Royal Infirmary University Hospitals Bristol NHS Foundation Trust Bristol UK; ^7^ Royal Papworth Hospital NHS Foundation Trust Cambridge UK; ^8^ The Christie NHS Foundation Trust Manchester UK; ^9^ Division of Surgery Royal Free London NHS Foundation Trust UCL London UK; ^10^ Derriford Hospital University Hospitals Plymouth NHS Trust Plymouth UK; ^11^ The Royal Wolverhampton NHS Trust New Cross Hospital Wolverhampton UK; ^12^ Surgical & Interventional Trials Unit (SITU) University College London London UK; ^13^ Centre for Trials Research Cardiff University Cardiff UK; ^14^ MRC Biostatistics Unit Cambridge UK; ^15^ Clinical Operational Research Unit University College London London UK

**Keywords:** Colorectal cancer, lung metastasectomy, randomized controlled trial

## Abstract

**Aim:**

Lung metastases from colorectal cancer are resected in selected patients in the belief that this confers a significant survival advantage. It is generally assumed that the 5‐year survival of these patients would be near zero without metastasectomy. We tested the clinical effectiveness of this practice in Pulmonary Metastasectomy in Colorectal Cancer (PulMiCC), a randomized, controlled noninferiority trial.

**Method:**

Multidisciplinary teams in 14 hospitals recruited patients with resectable lung metastases into a two‐arm trial. Randomization was remote and stratified according to site, with minimization for age, sex, primary cancer stage, interval since primary resection, prior liver involvement, number of metastases and carcinoembryonic antigen level. The trial management group was blind to patient allocation until after intention‐to‐treat analysis.

**Results:**

From 2010 to 2016, 93 participants were randomized. These patients were 35–86 years of age and had between one and six lung metastases at a median of 2.7 years after colorectal cancer resection; 29% had prior liver metastasectomy. The patient groups were well matched and the characteristics of these groups were similar to those of observational studies. The median survival after metastasectomy was 3.5 (95% CI: 3.1–6.6) years compared with 3.8 (95% CI: 3.1–4.6) years for controls. The estimated unadjusted hazard ratio for death within 5 years, comparing the metastasectomy group with the control group, was 0.93 (95% CI: 0.56–1.56). Use of chemotherapy or local ablation was infrequent and similar in each group.

**Conclusion:**

Patients in the control group (who did not undergo lung metastasectomy) have better survival than is assumed. Survival in the metastasectomy group is comparable with the many single‐arm follow‐up studies. The groups were well matched with features similar to those reported in case series.

## Background

The lung is a common site of metastases and, since the earliest days of chest radiography, has been the site where metastases are most easily detected. Their removal has been documented in case reports and in small follow‐up studies since the early days of thoracic surgery [1]. However, the claimed benefit of lung metastasectomy was challenged 40 years ago by Torkel Åberg. Introducing a small comparative study in 1980, he wrote, ‘It has been assumed, implied, or claimed that the 5‐year survival without operation is nil. Control material is, however, lacking’ [2]. His paper has rarely been cited [3].

Publication of the International Registry of Lung Metastases in 1997 was a landmark in the adoption of pulmonary metastasectomy [4]. The registry featured 5206 patients who had undergone a lung metastasectomy but included no information about patients who did not have a metastasectomy. The inherent assumption was that survival would have been negligible without resection of the metastases. This point is illustrated in a statement from a National Institute for Health and Care Excellence (NICE) guideline published in 2004: ‘Surgery for patients with metastases confined to the … lung … can improve five‐year survival from close to zero to over 30%’ [5]. This guideline is no longer accessible but was cited verbatim in an Analysis article in the BMJ in 2007 [6], pointing out the absence of evidence. The assumed low survival rate was re‐emphasized in an authoritative Rapid Response stating: ‘We have known the natural history of under‐treated metastatic colorectal cancer for over a decade’ [7].

The numbers of metastasectomy operations for colorectal cancer (CRC) grew between 2000 and 2011 without any controlled trials [8, 9], during a time when many randomized controlled trials (RCTs) of systemic therapies were conducted [10]. A literature search undertaken for a systematic review published in 2010 found 101 papers published on CRC lung metastasectomy [11]. None provided control data. This was the reason for running the Pulmonary Metastasectomy in Colorectal Cancer (PulMiCC) trial. Recruitment was poor, probably because of the entrenched belief that lung metastasectomy was life‐saving. The trial was stopped early, with analysable data available for 65 randomized patients [12]. Data on a further 28 randomized patients became available after the trial was published. An updated survival analysis is presented here, providing information on all 93 randomized patients at a date 18 months later than in the first publication.

Detection and treatment of metastases is central to the Impact Initiative (Improving Management of Patients with Advanced Colorectal Tumours) of the Association of Coloproctology of Great Britain and Ireland (ACPGBI) [13]. High among the research priorities considered in a modified Delphi approach is the question: ‘What is the optimal timing of resection of liver and/or lung metastases from colorectal cancer – before, during or after primary surgery?’ [14]. From the outset we make it clear that our paper concerns only lung metastases and the effect of metastasectomy on survival. Lung metastases considered for elective resection are asymptomatic so it is important to know how much survival benefit, if any, is actually gained because this is the motive for their removal.

## Method

A full account of the methods has already been published [12]. Below is an abbreviated version that includes the supplementary methods used for follow‐up of the additional patients and the new analysis.

### Study design

PulMiCC is a randomized Phase III, parallel‐arm, multicentre noninferiority trial conducted in hospitals treating advanced CRC. The principal investigators (PIs) were oncologists or surgeons working in multidisciplinary teams (MDTs). The randomized trial ran at 13 sites in England and one in Serbia.

The trial was coordinated initially by the Clinical Trials and Evaluation Unit, Royal Brompton and Harefield NHS Foundation Trust, London, and later by the Surgical and Interventional Trials Unit (SITU), University College London.

### Ethical approval and consent to participate

The National Research Ethics Service (NRES) granted ethical approval (no. 10/H0720/5) and recruitment began at each site after approval of local Ethics Committees. Written informed consent was obtained at enrolment and again at randomization (Stages 1 and 2, respectively). The trial protocol is available online (https://www.ucl.ac.uk/clinical‐operational‐research‐unit/sites/clinical‐operational‐research‐unit/files/pulmicc_protocol_december_2015.pdf).

### Patient participants

Adults who had resection of a CRC with a prospect of cure, but were found to have lung metastases, were recruited. There had to be no other sites of CRC other than treated liver metastases. The MDTs were required to have proven that these were CRC metastases or to have 90% clinical confidence that this was the diagnosis. Potential patients were invited to participate and gave initial consent to be monitored. If the MDT was uncertain as to whether a patient might or might not benefit from pulmonary metastasectomy, the patient was invited to enter the second phase of the study. If they consented, they were randomized either to metastasectomy or observation. The 419 who did not consent to be randomized continued to be monitored and that cohort will be the subject of a separate analysis.

### Randomization and masking

The randomization was to control or metastasectomy arms, with both arms similarly monitored. Patients were allocated equally, with stratification according to site. The sequence was generated at www.sealedenvelope.co.uk with minimization for age, sex, T(umour) stage, N(odal) stage, previous hepatic resection, interval since surgery for CRC, number of metastases and carcinogenic embryonic antigen (CEA) assay results, while retaining a random element. Minimization is largely a deterministic procedure that guarantees balance in stratifying factors and limits the potential for unexpected confounding [15, 16, 17]. The request and the assignment were communicated remotely, ensuring concealment from the trial centre and the sites. Masking at sites was deemed impossible but the assignment was not revealed to the trial management group until the analysis was completed.

Metastasectomy was performed by surgical resection, using either videothoracoscopy or open thoracotomy at the discretion of the surgeon.

Control patients were not treated initially with any local intervention, such as radiotherapy or image‐guided thermal ablation (IGTA).

### Outcomes

The primary outcome in the PulMICC trial was overall survival over the 5 years following from the date of randomization. Also reported in this paper is information on subsequent survival up to the date of the analysis.

### Data collection

The case report forms (CRFs) for patients in English centres were to be returned at 3, 6, 9, 12, 18, 24, 36, 48 and 60 months. Any treatment since the last report was recorded. Crossover effects were reflected in the as‐treated secondary analysis. For Serbian patients, data return had fallen into abeyance because of insurmountable difficulties at the time, and analysis was performed on the 65 available patients and published in 2019 [12]. Data were extracted from the standard CRFs returned at intervals as specified. Late or missing CRFs and missing fields were pursued by the Trials unit. Thereafter the PIs were contacted directly by the chief investigator (CI). Throughout this process the CI had no access to the assigned arms.

The CI was informed by the local PI on 24 November 2019 that the difficulties had been overcome. After exploration of the feasibility and likely completeness of the data, and with the agreement of the Chair of the Independent Data Monitoring Committee, patient‐specific CRFs requesting date of death or date last known to be alive, plus dates and nature of additional treatments for the Serbian patients, were sent out on 24 December 2019. These CRFs were returned on 21 January 2020 with a high level of compliance and completeness. Uncertainties or ambiguities concerning any of the 93 patients during this re‐analysis were resolved by exchanges of emails between the CI and the site PIs. Data entry for this second analysis was closed on 29 February 2020. Other information such as that from protocol‐determined lung function tests, were not available or retrievable and so was not requested.

### Statistical analysis

#### Sample size

A 10% difference in overall mortality at 3 years was taken to be the inferiority margin for the design of the PulMiCC noninferiority trial. A sample size of 1350 registered patients was estimated to provide 1:1 randomization of 300 patients.

#### Comparative analysis

For the primary outcome of survival, date of death and date last known alive were updated with a closing date of 29 February 2020. For comparative analysis, survival times were examined, and Kaplan–Meier estimates of survival curves were produced. Nonparametric CIs for survival times and quantiles were calculated using the R package ‘bpcp’ [18]. A Cox relative risk regression model [19], with an assumption of proportional hazards, was used to compare treatment arms in the intention‐to‐treat primary analysis in which a binary explanatory variable indicated treatment group. This provided estimated hazard ratios and confidence intervals. The assumption of proportional hazards was examined by testing for a linear trend of the treatment effect in time. The minimization variables were used for adjustment. For the as‐treated analysis, comparison was based on a time‐dependent binary explanatory variable, which was zero until the time at which a metastasectomy occurred, when it took a value of 1 [12]. Crossovers are thus accounted for in this analysis.

## Results

The first randomization was 2 December 2010 and the last was 24 November 2016. The randomized trial closed in December 2016 because of poor recruitment. There were 512 patients in Stage 1, of whom 93 were randomized. The PIs, clinical sites and numbers randomized at each site are listed in Table 1.

**Table 1:** Principal investigator, sites and numbers of randomized patients.

Principal investigator	Clinical sites	Randomizations
Misel Milosevic	Thoracic Surgery Clinic, Institute for Lung Diseases of Vojvodina, Sremska Kamenica, Serbia	28
John Edwards	Sheffield Teaching Hospitals NHS Foundation Trust, Sheffield, UK	18
David Tsang	Basildon and Thurrock University Hospitals NHS Foundation Trust, Basildon, UK	8
Joel Dunning	The James Cook University Hospital, South Tees Hospitals NHS Foundation Trust, Middlesbrough, UK	7
Michael Shackcloth	Liverpool Heart and Chest Hospital NHS Foundation Trust, Liverpool, UK	7
Tim Batchelor	Bristol Royal Infirmary, University Hospitals Bristol NHS Foundation Trust, Bristol, UK	5
Aman Coonar	Royal Papworth Hospital NHS Foundation Trust, Cambridge, UK	5
Jurjees Hasan	The Christie NHS Foundation Trust, Manchester, UK	4
Brian Davidson	Royal Free London NHS Foundation Trust, London, UK	3
Adrian Marchbank	Derriford Hospital, University Hospitals Plymouth NHS Trust, Plymouth, UK	2
Simon Grumett	New Cross Hospital, The Royal Wolverhampton NHS Trust, Wolverhampton, UK	2
Eric Lim	Royal Brompton Hospital, Royal Brompton & Harefield NHS Foundation Trust, London, UK	2
Apostolos Nakas	Glenfield Hospital, University Hospitals of Leicester NHS Trust, Leicester, UK	1
Stelios Vakis	Queen's Hospital, University Hospitals of Derby and Burton NHS Foundation Trust, Burton upon Trent, UK	1
**Total randomized**		**93**

The total randomized (*n*=93) represents 18% of the total of 512 patients recruited to Pulmonary Metastasectomy in Colorectal Cancer (PulMiCC).

The dataset was closed on 29 February 2020, adding 18 months of follow‐up for the UK patients in the previous report [12] as well as available information on survival for the Serbian patients. The median follow‐up for all patients was 3.46 years compared with 3.16 years in the previous report. The mean follow‐up for patients alive at the last follow‐up was 4.51 years compared with 3.85 in the previous report. For all but two patients, follow‐up was longer than 3 years, and 81 had been followed up for longer than 5 years or had died before this time. Of the 93 patients randomized, 47 were assigned to the control group and 46 to metastasectomy. No patient had metastasectomy or stereotactic body radiotherapy (SBRT) to the index metastasectomy site in the first year. The clinical teams were subsequently allowed to treat as they judged clinically appropriate, and three patients in the control group had metastasectomy at 13, 19 and 27 months after randomization. Three patients assigned to metastasectomy did not have it: two preferred not to have an operation; and one was found to have progressing brain involvement. One patient in each arm turned out not, in fact, to have metastases. In one control group patient with presumed metastases, the opacities had resolved on a CT scan 5 months later. The patient remains alive after 9 years. A patient assigned to metastasectomy had two nodules removed, which were found to be intrapulmonary lymph nodes, and this patient remains alive 7.5 years later. Both patients remain in their assigned group for intention‐to‐treat analyses.

There were no treatment‐related deaths or major adverse events. It should be noted that in the context of thoracic surgery these are among the least hazardous lung operations. Because of the highly selective nature of the practice, unlike with lung cancer surgery, higher‐risk operations can be avoided.

**Figure 1:**
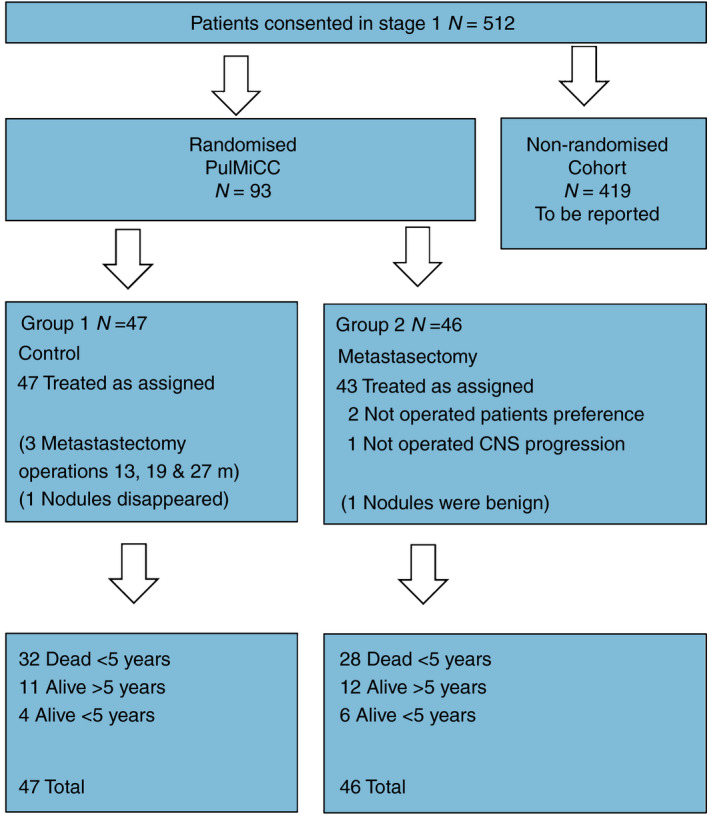
Consort flow diagrams of the randomized trial. CNS, central nervous system; m, months; PulMiCC, Pulmonary Metastasectomy in Colorectal Cancer.

**Figure 2:**
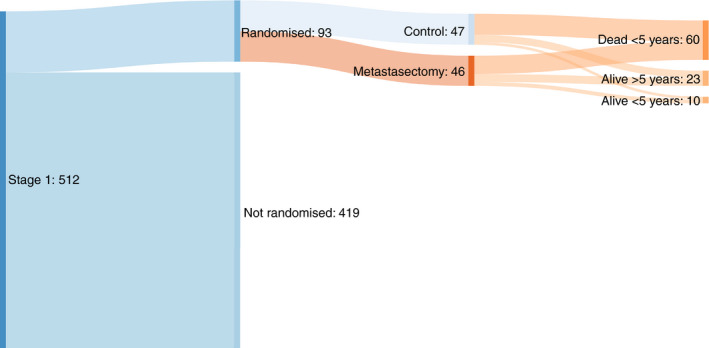
Sankey flow diagram of trial outcomes. y, years.

Table 2 shows the balance in minimization variables between the two treatment groups. Table 3 shows the distributions of age, gender, CRC resection interval and number of metastases for the two groups. Figure 1 gives a profile of the patients in the PulMICC trial. Figure 2 is a Sankey chart illustrating the flow of patients. [http://sankeymatic.com/faq/].

**Table 2:** Data obtained at baseline in all patients and used in the minimization step in randomizing patients to the two trial arms.

Characteristic	Group 1 (*N* = 47)	Group 2 (*N* = 46)
	Control	Metastasectomy
Gender	
Male	28	31
Female	19	15
Age (years)	
61+	33	32
60 or under	14	14
Lung metastases	
1	16	18
2–4	26	24
5+	5	4
CEA (ng/ml)	
<5	36	37
5–10	6	6
10+	5	3
Prior liver resection	
Yes	13	14
No	34	32
Years since 1^o^ CRC resection	
<1	7	7
1–3	28	26
3+	12	13
CRC Stage	
T stage	
1	2	2
2	8	7
3+	37	37
N Stage	
0	25	24
1+	22	22

Values represent the number of patients.

CEA, carcinoembryonic antigen; CRC, colorectal cancer.

**Table 3:** Distributions of all patients, stratified according to gender and arm to which they were assigned at randomization (top).

Characteristic		Minimum	25%	50%	75%	Maximum
Age (years)					
Male					
Control	*N* = 27/27	55.4	62.4	68.5	74.2	86.5
Metastasectomy	*N* = 31/31	35.3	58.5	66.4	72.1	82.8
Female					
Control	*N* = 20/20	48.2	54.3	61.3	74.3	83.2
Metastasectomy^†^	*N* = 14/15	50.8	64.4	71.6	64.4	76.5
CRC resection interval (months)					
Control^‡^	*N* = 46/47	2.0	17.2	27.4	35.0	130.5
Metastasectomy^§^	*N* = 45/46	1.0	13.8	23.1	36.8	106.5
Number of metastases					
Control^¶^	*N* = 46/47	1	1	2	3	8
Metastasectomy	*N* = 46/46	1	1	2	3	6

Values are given as minimum, maximum and quantiles. The quantile distribution of the number of metastases is as follows: for 1, 2, 3, 4 and 5 metastases the exact numbers for control patients were 16, 16, 7, 3 and 3, and for metastasectomy patients they were 16, 17, 8, 2 and 1, with one patient in each arm having more than 5 metastases – 8 and 6, respectively.

^†^ We know from minimization data that the age category of the missing patient was 61+ years.

^‡^ From minimization data, the missing colorectal cancer (CRC) resection interval [i.e., the time elapsed between the primary CRC resection and the metastasectomy operation] was 1–3 years.

^§^ The missing CRC resection interval was <1 year.

^¶^ From minimization data, the metastasis count was >5.

### Additional treatments

There were no significant differences in the intensity of other treatments which might have altered the balance between the groups.

The intention of the CRF question was to capture treatment of lung metastases but in one instance in each group the reported radiotherapy was to treat metastases elsewhere (brain and bone). However, there is no evidence that patients were treated with radiotherapy to the index lesion if they were assigned to the non‐metastasectomy group. The only IGTA used was radio frequency ablation (RFA): In addition to the treatment in the table, one patient in each group and repeated RFA to a total of 3 treatments in each case.

Additonal treatments are in Table 4.

**Table 4:** Additional treatments.

(a) Numbers of patients and cycles of chemotherapy					
Group	*N*	Treated	Cycles	Median	Total cycles
Control	47	23 (49%)	1–6	3	68
Metastasectomy	46	19 (41%)	2–7	3	60

(b) Timing from randomisation in months						
Group	*N*	< 6*	6–12	Earliest	Median	IQR
Control	47	9	3	0.4	11.6	2.3–16.4
Metastasectomy	46	6	7	1.0	7.8	5.4–14.1

(c) Radiotherapy and IGTA in months					
Radiotherapy	*N*	< 6	6–12	> 12	Total
Control	47	0	1	3	4
Metastasectomy	46	0	1	4	5

IGTA	*N*	< 6	6–9	9–12	> 12	Total
Control	47	0	1	1	1	3
Metastasectomy	46	0	1	1	0	2

* Any differences may in part reflect considerations of fitness for chemotherapy in the post‐operative period.

### Survival

#### Updated primary trial outcome analyses

**Figure 3:**
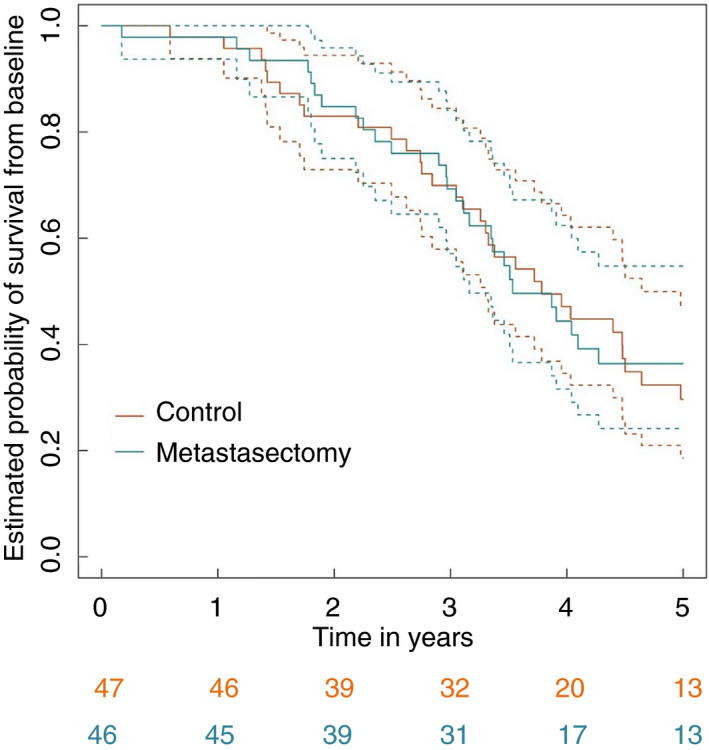
Kaplan–Meier survival curves for control and metastasectomy arms.

Restricting attention to 5 years of follow‐up, as specified for the primary analysis of the trial, 58 deaths (31 in the control group and 27 in the metastasectomy group) were recorded at the close of the analysis. Figure 3 presents a Kaplan–Meier estimate of the survival curves for the control and metastasectomy arms.

Comparison of survival rates in the metastasectomy arm with those in the control arm, adjusting for and therefore comparing patients with comparable minimization variables, gave an estimated hazard ratio of 0.87 with a 95% CI of 0.51–1.48. There was no evidence for a nonproportional hazard (*P* = 0.47). The unadjusted estimated hazard ratio was 0.93 (95% CI: 0.56–1.56). For the ‘as‐treated’ analyses, the comparable adjusted and unadjusted estimated hazard ratios were 0.73 (95% CI: 0.42–1.28) and 0.81 (95% CI: 0.48–1.37).

#### Complete survival data

Over the entire follow‐up period, 63 deaths were recorded: 33 in the control arm and 30 in the metastasectomy arm. Table 5 presents the observed minimum and maximum survival times in both arms and the estimated nonparametric 25%, 50% (median) and 75% quantiles of the time‐to‐death distributions along with associated 95% CIs. The tabulated values are very similar in both arms, with differences only seen in the 75% quantiles, which are estimated from very limited data. The median survival after metastasectomy was 3.5 (95% CI: 3.1–6.6) years compared with 3.8 (95% CI: 3.1–4.6) years for controls.

**Table 5:** Survival time (in years) for all patients, from randomization.

		Survival quantiles				
Patient group		Min.	25% (95% CI)	50% (95% CI)	75% (95% CI)	Max.
Control	Group 1 (*N* = 47)	0.59	2.74 (1.53–3.32)	3.78 (3.10–4.64)	5.63 (4.48 to Inf)	9.05
Metastasectomy	Group 2 (*N* = 46)	0.17	2.90 (1.83–3.35)	3.53 (3.11–6.58)	7.46 (4.10 to Inf)	9.07

Inf, Infinity.

Overall estimated survival at 4 years was 47.1% (95% CI: 31.9%–62.6%) for control patients and 44.4% (95% CI: 28.8%–60.6%) for metastasectomy patients, with the respective 5‐year survival values being 29.6% (95% CI: 15.3%–45.7%) and 36.4% (95% CI: 21.3%–53.0%). The estimated 4‐year survival percentages are closer than reported previously, and the 5‐year percentages are comparable [12]. There is a numerical difference in estimated 5‐year rates because there were seven (of 20) deaths in the control arm in year 5 and three (of 17) in the metastasectomy arm. Note, however, that the deaths in the metastasectomy arm occurred earlier in year 5 than those in the control arm. Subsequently, there were three and two deaths in the two arms, respectively.

Respiratory function and Patient Reported Outcome Measures were reported in 2019 for 65 randomised patients [12]. There are no further data to report here.

## Discussion

The main limitations of the PulMiCC trial are small numbers and early closure [12]. This update increases the numbers by 43% and adds 18 months of follow‐up information for all patients. The 5‐year survival in the well‐matched control group is 29.6% (95% CI: 15.3%‐45.7%). This undermines the ‘close to zero’ assumption for the survival of patients with CRC lung metastases without lung metastasectomy.

Power calculations are based on the most reliable data available when planning the study. The power calculation for PulMiCC was not based on a ‘close to zero’ assumption because a modelling study based on UK cancer registry data, carried out in 2008, had indicated that 5‐year survival in the control group was likely to be much higher than assumed [20]. After careful statistical consideration, we based the power calculation on finding a difference, of less than 10%, in overall mortality at 3 years, assuming 3‐year survival in the metastasectomy group of 30%, substantially less than actually observed. Small differences require large numbers and so the power calculation required 300 patients to have sufficient expected information to examine a 10% noninferiority margin for continued active monitoring compared with metastasectomy.

There are occasional patients reported or remembered who survive a long time and in whom lung metastases eventually appear to have been the only cancer remaining. However, in this controlled trial most patients went on to die of disseminated cancer at a similar rate, regardless of whether they did or did not have lung metastasectomy. This attrition is also seen in observational reports. The results of PulMiCC are inconclusive for the main intended outcome, but the finding of a much higher‐than‐expected survival of the control patients, compared with what is assumed in nonrandomized (observational) studies, is important.

We know of two other randomized trials in which the effectiveness of local treatment of metastases was tested, both of which also found higher survival in the control group than expected by the trialists. The CLOCC trial tested RFA for liver metastases and SABR‐COMET tested stereotactic radiotherapy for any primary site and any secondary site (except brain). The 5‐year survival in the treated arms was 40%–45%, as reported for lung metastasectomy, but 5‐year survival was 30% and 25% in the control groups, similar to the 30% reported here for PulMiCC control patients. Survival without metastasectomy in CLOCC and PulMiCC taken together, a combined total of 106 patients with CRC metastases, was 30% (95% CI: 21%‐40%) derived using a complementary log‐log scale. The authors of CLOCC wrote that ablation of metastases ‘results in an excellent survival, which however was also achieved in the control arm’ [21] and the authors of SABR‐COMET commented that the ‘better‐than‐expected survival in both groups suggest that oligometastatic cancers behave more indolently than previously appreciated’ [22]. They had been misled by the ‘close to zero’ assumption. Also it should be noted that in both of these trials (CLOCC and SABR‐COMET) there were imbalances in the numbers of metastases between the arms, which favoured the interventional arm [23, 24]. The number of metastases is a powerful prognostic fact with a hazard ratio for multiple *vs* solitary CRC lung metastases of 2.04, and so it would have been better to ensure balance in this factor [25]. In SABR‐COMET, there was an additional imbalance in cancer types, also favouring the interventional arm. But it is the very similar and better‐than‐expected survival in the control arms of all three trials that is important to note.

So where did the assumption of very poor survival in untreated patients come from? The NICE source [5, 6, 7] can be traced back to a 1994 paper confined to a retrospective analysis of liver resection [26]. Patients deemed inoperable had poor survival but they bear no resemblance to candidates for lung metastasectomy. Then, in a systematic review of CRC lung metastasectomy in 2013, the assumption was moderated to ‘5‐year survival rates worse than 5%’ [25]. Cited in support is a 1989 publication comparing five different methods of delivering fluorouracil to CRC patients who had characteristics far worse than candidates for lung metastasectomy [27]. The few papers that address the question of survival rates without metastasectomy use the <5% assumption, referring to the systematic review or other secondary or unsubstantiated sources. The Society of Thoracic Surgeons (STS) Work Force of Evidence Based Surgery subjected pulmonary metastasectomy to an STS expert consensus development process. Their publication, in 2019, cites 167 papers and they comment that: ‘metastatic disease survival is assumed to be zero, a contention not supported by the literature’ [28]. The current NICE guideline states that lung metastasectomy should be ‘considered’ but provides no good evidence of effectiveness [29].

If control 5‐year survival was indeed 5%, an RCT in 40 patients could have shown the large difference believed to be gained by lung metastasectomy. If survival in the metastasectomy group was expected to be 40%, then the power to detect an effect in a trial with 20 patients in each arm would be 83%. Large effects, where they exist, can be shown with small trials. The feasibility of such a trial was considered at the Mayo Clinic in 1992, with a similar estimate of the required power, but no trial was carried out [30, 31].

High on the list of research priorities of the ACPGBI is the question ‘Can early markers of metastatic disease be developed?’ [14]. There is ample evidence that earlier detection of metastases does not lengthen survival. A systematic review found 16 RCTs comparing standard with more intensive surveillance in patients treated for early CRC. Meta‐analysis of 11 RCTs with adequate data found that more intensive monitoring advanced detection of metastases by a median of 10 months. As a result, there were more metastasectomy operations, but no resulting survival benefit [32]. In fact, in the largest three RCTs included (33, 34, 35), there was an adverse effect on survival despite higher detection. These findings were confirmed by a separately conducted Cochrane review and meta‐analysis [36]. The British Journal of Surgery’s editor regarded the conclusion as ‘bleak nihilism’ and wrote ‘it is counterintuitive that earlier identification of metastatic disease does not improve survival’ [32], an opinion counter to evidence. Uncertainty about the yield from metastasectomy was expressed by the authors of a meta‐analysis of CRC survival gains who noted ‘that while indeed more metastasectomies are being performed, they have been made possible by better therapies and that this benefit should be ascribed to the therapies’. This raises the possibility of reverse causation [10] – longer survival providing opportunities for more treatments rather than additional treatments necessarily resulting in longer survival.

In view of all these uncertainties and the results of PulMiCC, the widespread belief in the value of metastasectomy needs to be challenged. Large, definitive randomized trials, investigating the possible benefits of the practice of pulmonary metastasectomy for any tumour type, are clearly needed and should be based on the realistic survival figures that three small randomized trials have provided. Meanwhile, the results of PulMiCC should inform clinical practice, and patients who are offered metastasectomy (whether surgical or by ablation) should be clearly told about the uncertain benefits and possible risks.

## Conflicts of interest

None of the authors has a conflict of interest with respect to any of the content of this submission.

## Consent for publication

Not applicable. No patient information is disclosed.

## Ethics approval

Ethics approval was granted by the National Research Ethics Committee London – Hampstead 10/H0720/5.

## Data Availability

All information is freely available by application to the Chief Investigator TT and SITU UCL.
