# Supplementary material for: Pulmonary Metastasectomy in Colorectal Cancer: updated analysis of 93 randomized patients – control survival is much better than previously assumed
Source: Colorectal Dis. 2020 Jun 14;22(10):1314–24. doi: 10.1111/codi.15113 (PMC7611567; doi:10.1111/codi.15113)
Supplement: Supplementary file 1 [file CODI-22-1314-s001.docx]

Table 1

|  | Group 1 N=47 | Group 2 N=46 |
| --- | --- | --- |
|  | Control | Metastasectomy |
| Gender | X (%) | X(%) |
| Male | 28 (60) | 31 (67) |
| Female | 19 (40) | 15 (33) |
| Age (years) |  |  |
| 61+ | 33 (70) | 32 (70) |
| 60 or under | 14 (30) | 14 (30) |
| Lung metastases |  |  |
| 1 | 16 (34) | 18 (39) |
| 2 to 4 | 26 (55) | 24 (52) |
| 5+ | 5 (11) | 4 (9) |
| CEA ng/mL |  |  |
| <5 | 36 (77) | 37 (80) |
| 5-10 | 6 (13) | 6 (13) |
| 10+ | 5 (11) | 3 (7) |
| Prior liver resection | |  |
| Yes | 13 (28) | 14 (30) |
| No | 34 (72) | 32 (70) |
| Years since  1^o^ CRC resection |  |  |
| <1 | 7 (15) | 7 (15) |
| 1 to 3 | 28 (60) | 26 (57) |
| 3+ | 12 (26) | 13 (28) |
| CRC Stage |  |  |
| T stage |  |  |
| 1 | 2 (4) | 2 (4) |
| 2 | 8 (17) | 7 (15) |
| 3+ | 37 (79) | 37 (80) |
| N Stage |  |  |
| 0 | 25 (53) | 24 (52) |
| 1+ | 22 (47) | 22 (48) |
